# Strategic role of poultry production sciences in shaping the future of global food security and strengthen sustainability

**DOI:** 10.1016/j.psj.2026.106617

**Published:** 2026-02-10

**Authors:** Farid S. Nassar

**Affiliations:** Department of Animal and Fish Production, College of Agricultural and Food Sciences, King Faisal University, Al-Ahsa, Saudi Arabia

**Keywords:** Poultry production sciences, Food security, Sustainability, Consumer behavior, Social transformations

## Abstract

Poultry production sciences play a strategic role in enhancing food security and meeting the growing global demand for animal protein, while also contributing to sustainability. Understanding and leveraging consumer behaviors enables the development of flexible and sustainable production systems capable of effectively adapting to social changes and future market needs. This study aims to explore the strategic role of poultry production sciences in shaping the future of global food security and achieving sustainable development. A descriptive methodology was adopted, based on a systematic review of peer-reviewed literature, international reports from relevant organizations, and an analysis of global case studies. Studies were included if they examined the role of poultry production in meeting consumer demands and promoting sustainability. Studies not related to higher education or production contexts, or lacking methodological rigor or empirical evidence, were excluded. The findings indicate that integrating scientific research, applied education, and insights into consumer behavior enables poultry science programs to align graduates’ skills with market demands, optimize production strategies, and enhance innovation, resilience, and sustainability across the sector. Additionally, Poultry science programs are a fundamental driver of scientific and technological advancement, linking research outcomes with market trends and consumer behavior, developing specialized human capital, and supporting evidence-based policymaking to enhance the resilience of global food systems. The study also emphasizes the importance of understanding consumer behavior as a strategic tool that enables producers, decision-makers, and agricultural policymakers to translate these insights into innovative and sustainable production practices, thereby boosting competitiveness, meeting future demand, and maintaining a balance between profitability, quality, and social and environmental responsibility. Moreover, the study recommends fostering collaboration among policymakers, producers, and academic institutions through joint initiatives and measurable monitoring systems to develop forward-looking strategies and awareness programs, thereby embedding resilience and sustainability as core pillars for the long-term advancement of the global poultry industry.

## Introduction

By 2050, the global population is projected to reach around 9.8 billion, which is expected to double the demand for poultry products due to urbanization, demographic shifts, and rising living standards. The poultry industry, with its high efficiency in converting feed into nutrient-dense protein, plays a crucial role in supporting local food security and alleviating poverty (Kleyn and Ciacciariello, 2021). Ensuring long-term sustainable growth requires aligning production systems with environmental, social, and economic sustainability standards, while also raising consumer awareness and encouraging purchasing behaviors that favor more sustainable dietary choices, thereby contributing to a balanced and resilient global food system (Kleyn and Ciacciariello, 2021).

In November 2022, the United Nations reported that the global population had reached 8 billion, reflecting a remarkable increase from 2.5 billion in 1950 (United Nations, 2022). This extraordinary demographic growth has substantially escalated the global demand for food, especially for poultry products. Promoting sustainability in poultry production requires a comprehensive approach that ensures the well-being of the environment, the animals, the producers, and the communities that rely on this critical industry for their livelihoods (Conrad et al., 2018).

Population growth, urbanization, and rising incomes have positioned chicken meat as a preferred protein source for many consumers due to its affordability, low fat content, and compatibility with cultural and religious dietary practices (Dewi et al., 2024). In high-income countries, consumer preference is largely driven by convenience and perceived health benefits, whereas in low- and middle-income countries, its appeal is primarily linked to its lower cost relative to other sources of animal protein (OECD/FAO, 2023). As a result, ensuring adequate and sustainable poultry production to meet the escalating global demand has become essential. Achieving sustainability in this sector requires reinforcing the three pillars, social, economic, and environmental, with consumers playing a critical role, as their choices and purchasing behaviors frequently influence industry practices and priorities (Dewi et al., 2024).

Moreover, chicken is the most widely consumed type of meat globally due to its affordability, ease of preparation, versatility, and mild flavor, making it suitable for diverse diets without major religious, ethical, or environmental constraints. It is also considered low emission meat, making it a preferred choice for environmentally conscious consumers and those following flexitarian diets. With an average annual consumption of 15.7 kg per person, poultry serves as a reliable and essential source of protein, playing a key role in meeting nutritional needs worldwide while remaining accessible and cost-effective (Viktória and Zoltán, 2023).

The inherent advantages of poultry meat are expected to sustain its consumption even amid evolving consumer behaviors (Jez et al., 2011). Analyzing consumer preferences and market trends allows stakeholders and policymakers to identify emerging patterns and potential challenges, enabling them to anticipate impacts and implement proactive strategies (Jez et al., 2011). Strengthening the poultry sector by highlighting its social acceptability, relative ease of management compared to other livestock industries, and economic potential can serve as a key strategic objective. Achieving a balanced and sustainable approach requires a thorough examination of factors such as breeding and slaughtering practices, genetic potential, processing technologies, and other related elements (Jez et al., 2011).

The established perception of poultry as a healthy, convenient, and affordable protein source continues to drive its widespread consumption (Dewi et al., 2024). These insights also provide producers with a basis for enhancing their understanding of consumer attitudes, priorities, and the motivations or barriers influencing purchasing behavior. Such knowledge is critical for designing strategies that encourage the adoption of sustainable production practices (Dewi et al., 2024). By identifying the specific attributes that consumers value, stakeholders can make informed decisions on which practices to implement and how to effectively communicate these changes to gain public approval and financial support. Furthermore, increasing consumer awareness of poultry sustainability is essential, as it shapes acceptance and willingness to absorb any additional costs associated with sustainable production (Dewi et al., 2024).

In light of the pressing challenges facing global food security and the rapid growth of the world population, poultry science programs play a strategic role in shaping a sustainable future for the sector. They integrate academic education, practical training, and scientific research to develop advanced, efficient, and sustainable poultry production systems capable of meeting the increasing nutritional needs of consumers at both local and global levels (Nassar and Abbas, 2025a). These programs contribute to enabling the industry to improve resource-use efficiency and promote environmentally sustainable practices (Nassar and Abbas, 2025a; Nassar and Abbas, 2025b). Moreover, they support innovation in developing healthy, safe, and environmentally friendly poultry products, thereby enhancing global food security and advancing sustainable development, positioning the poultry sector as a cornerstone for a secure and sustainable food future (Nassar and Abbas, 2025a; Nassar and Abbas, 2025b).

This study aims to develop a scientific and practical framework that enables the critical poultry sector to address current challenges and meet future demands. In this context, the study seeks to address four primary areas: first, to identify the strategic significance of poultry production in meeting the global demand for animal protein amid population growth, highlighting the sector’s vital role in global food security. Second, to explore technological advancements in the poultry industry that enhance production efficiency and promote sustainability, as modern innovations play a key role in improving performance while minimizing environmental impact. Third, to examine how consumer behaviors and preferences influence poultry production, linking actual demand with sustainable production practices. Fourth, to analyze the strategic role of poultry science programs in shaping the future of global food security and achieving sustainability, through the integration of academic education, practical training, and scientific research to prepare professionals capable of addressing future challenges.

Poultry production sector constitutes a cornerstone of food security at both national and global levels, providing a vital source of animal protein and playing a pivotal role in meeting the increasing nutritional demands resulting from global population growth. In this context, the strategic role of poultry science programs becomes critically important, as they contribute to shaping a sustainable and resilient future for the sector—one that balances rising demand with environmental and social sustainability.

One of the most effective mechanisms for achieving this role is the integration of advanced academic education, practical field training, and applied scientific research within poultry science programs. These programs aim to develop advanced, efficient, and sustainable production systems, focusing on optimizing resource utilization, reducing waste and carbon emissions, implementing environmentally responsible practices, and fostering innovation in the production of healthy, safe, and environmentally friendly poultry products.

Despite the abundance of studies addressing technical and economic aspects of poultry production, a clear gap exists in the literature regarding the strategic role that poultry science programs must play in preparing the sector to adapt to future social transformations and evolving consumer behaviors. Most studies have focused separately on production efficiency, environmental impact, or consumer trends, without providing a comprehensive, forward-looking analysis of how these programs can enhance the industry’s capacity to strategically respond to societal and value-based changes, and emerging trends in animal protein consumption, thereby strengthening sector resilience, sustainability, and its future role in global food security.

Ultimately, poultry science programs play a strategic role in building a sector capable of addressing future challenges with confidence and efficiency, achieving sustainable production, supporting global food security, and promoting environmental, economic, and social sustainability. Through these programs, the poultry sector becomes a driving force for innovation and sustainable development, contributing to the creation of a safe and sustainable food future for all.

## Material and methods

This study employs a qualitative descriptive research design to investigate the strategic role of poultry production sciences in shaping the future of global food security and promoting sustainability. As Neuman (2014) explains, descriptive research is characterized by its capacity to “provide an accurate and detailed depiction of a particular situation, social context, or relationship” and to begin with a clearly defined question, aiming to describe it precisely, while also addressing the “how” and “who” of the phenomena under study. Moreover, Lambert and Lambert (2012) further emphasize that qualitative research designs, including phenomenology and grounded theory, can serve both descriptive and explanatory purposes. They argue that labeling a study as “qualitative descriptive research” offers a more precise classification than associating it with other qualitative methodologies, such as phenomenology, grounded theory, or ethnography. Moreover, qualitative descriptive research “often originates from naturalistic inquiry”, seeking to examine phenomena in their natural context as authentically as possible. In addition, aligned with its qualitative approach, this study aims to explore how poultry production sciences contribute strategically to enhancing global food security and achieving sustainability, situating it firmly within the qualitative descriptive paradigm. To address the research objectives, a comprehensive content analysis was conducted across both electronic and printed sources pertinent to the topics of interest (Bowen, 2009). Bowen (2009) outlines three principal stages in content analysis: skimming, careful reading, and interpretation, enabling systematic organization of textual data into smaller, meaningful units (Weber, 1990). This process facilitates the identification of recurring themes and patterns, allowing the study to uncover underlying meanings across multiple sources (Patton, 2002).

Based on an extensive review of existing literature and online resources, this study employed a structured four-phase methodology to provide a thorough and precise analysis of the strategic role of poultry production sciences. Phase One involved a systematic and comprehensive search across leading academic databases, including Google Scholar, Scopus, and Web of Science. A targeted set of keywords such as “Poultry Production,” “Poultry Science Programs,” “Higher Education,” “Chicken Meat Consumers,” “Sustainability,” “Poultry Products,” “Future Poultry Consumption,” and “Future Food Security” was used to identify relevant sources. The search focused on peer-reviewed articles, institutional reports, and empirical studies published in English over the past five years, supplemented by foundational literature to establish a robust theoretical framework. Moreover, Phase Two entailed defining clear inclusion and exclusion criteria. Studies were included if they directly addressed the role of poultry production in meeting current and future consumer demands while promoting sustainability. Research not explicitly linked to higher education or production environments, or lacking methodological rigor or empirical evidence, was excluded.

In phase three, involved the extraction and analysis of data from the selected sources. The analysis focused on each study’s objectives and key findings regarding poultry production’s contribution to satisfying current and projected consumer needs and advancing sustainability. The qualitative examination explored consumer behavior, anticipated future demands, and critical influencing factors, with the aim of identifying how poultry science programs can utilize these insights to develop sustainable production systems capable of efficiently meeting growing demand. Phase Four, the final stage, comprised an in-depth synthesis of findings to examine the strategic role of poultry production sciences in shaping the future of global food security and sustainability within an integrated framework. This phase emphasized the initiatives and training programs that higher education institutions and poultry science curricula should implement to maximize their contribution to global food security and long-term sectoral sustainability.

Moreover, throughout all phases of information handling, the retrieved materials were systematically screened for relevance, then organized using a structured information-extraction framework (including study title, abstract, results, and key findings) and managed through thematic coding and categorization procedures. Subsequently, the information was analyzed using qualitative thematic analysis to identify recurring patterns, key themes, and emerging trends, with findings synthesized and interpreted in alignment with the study’s conceptual framework and research objectives, ensuring methodological rigor.

This integrated methodological framework provides a comprehensive and precise assessment of the role of poultry science programs in fostering a sustainable future for the industry, enhancing their capacity to address forthcoming challenges, and ensuring the resilience of global food security over the long term.

## Results and discussion

### Strategic significance of poultry production in meeting the global demand for animal protein in the context of population growth

The strategic importance of poultry production lies in its ability to ensure global food security, provide high-quality and accessible animal protein, and support economic stability. Its efficiency, flexibility, rapid production cycles, and integration with modern supply chains make it a key driver of sustainable food systems, while also strengthening rural economies, supporting livelihoods, and creating opportunities for value-added products to meet current and future nutritional needs and address population growth.

In this context, poultry has emerged as the preferred source of protein for millions of consumers worldwide (Ashrafudoulla et al., 2021). In 2019, global chicken meat production accounted for approximately 40 % of total meat production, making it the most widely produced meat globally (FAO, 2021). With the continued growth of the world population, this trend is expected to persist. By 2030, poultry meat consumption is projected to reach 145 million metric tons, an increase of 15 million tons compared to 2022, representing roughly half of the anticipated growth in total meat production (OECD/FAO, 2021). In this context, the poultry industry plays a crucial role in meeting future consumer demand (Chowdhury et al., 2023).

This growing demand is closely linked to the rapid expansion of the global population, which is expected to increase by 2 billion people over the next 27 years, reaching an astonishing 9.7 billion by 2050 (United Nations, 2019). This population growth drives higher food demand and the expansion of food supply. The poultry industry stands out as a key food source among various food suppliers. In 2020, poultry meat accounted for approximately 40 % of total global meat production over the past three decades (FAO, 2023).

Based on these global dynamics, Poultry meat is currently experiencing its golden era, both locally and globally. Its beneficial physiological effects, high nutritional value including its rich protein content, and vital role in modern diets have made it the most popular and widely consumed type of meat today (Viktória and Zoltán, 2023). The global health-conscious trend has further fueled the demand for poultry meat, a trend that has persisted for decades, as reflected in the continuous growth of its production, trade, and consumption. Moreover, the shift toward environmental awareness has further strengthened the poultry market, as poultry meat is among the least environmentally impactful animal protein sources (Viktória and Zoltán, 2023). In the European Union, poultry ranks as the second most consumed type of meat; however, in several European countries, it has already become the leading meat in total consumption. Poultry meat is valued not only for its health benefits and low environmental footprint but also for its convenience, versatility in preparation, quick cooking time, and affordability as a protein source. These combined advantages are expected to drive further growth in production, trade, and consumption in the coming years, with China, the United States, the European Union, and Brazil continuing to dominate the global poultry sector (Viktória and Zoltán, 2023).

Aligned with this upward trend, global poultry meat production is projected to rise by 2 % in 2025, reaching approximately 105.8 million tons, driven by increased output from major producers such as the United States, the European Union, Brazil, Turkey, and China. Although corn and soybean meal prices remain relatively high, the anticipated decline in feed costs is expected to boost production across most countries (USDA, 2025). In the European Union, production is forecast to grow by 2 %, supported by strong domestic demand and reduced imports from Ukraine, as well as lower energy and feed costs that have enhanced producer profitability, particularly in Poland, the EU’s leading producer. Meanwhile, Brazil’s production is projected to reach a record 15.3 million tons, up 2 %, fueled by improved profit margins, sustained strong export demand, and rising domestic consumption driven by population growth (USDA, 2025).

However, the interplay between population growth and consumption patterns continues to shape the sector’s future. Population growth leads to a continuous increase in food demand (United Nation, 2021; FAO, 2022), coupled with rising per capita income, which further strains natural resources and biodiversity (Foley et al., 2011). Meat consumption in Europe is expected to rise slightly over the next decade (Statista, 2020; EC, 2021), with only shifts occurring in consumers’ preferred types of meat (EC, 2022A). Poultry is the only meat category projected to see increased consumption (OECD/FAO, 2022), primarily in the chicken sector, followed by turkey. Health concerns, animal welfare, and environmental protection are also driving higher poultry consumption (Statista, 2020). Among recent trends, the growing adoption of plant-based diets has expanded the number of flexitarians, vegetarians, and vegans (Statista, 2020), which may represent the most significant constraint on poultry meat consumption (Viktória and Zoltán, 2023).

At the level of global poultry meat consumption, intake is projected to reach 173 million tons ready-to-cook by 2034, representing 62 % of the total increase in global meat consumption. The growth in poultry consumption over the past decade has been largely driven by rising demand in Asia, particularly in China, India, Indonesia, Pakistan, and Vietnam, and this trend is expected to continue. Rapid consumption growth is also anticipated in regions such as Brazil, Egypt, Mexico, the Philippines, and the United States (OECD/FAO, 2025). Moreover, the global expansion of poultry-derived protein has accounted for an increasing share of total meat protein intake, a defining feature of meat consumption growth in recent decades, and this trajectory is expected to persist. By 2034, poultry is projected to provide approximately 45 % of the total protein obtained from all meat sources. This growth is primarily attributed to its affordability, as poultry remains the most economically accessible meat, and its favorable nutritional profile, offering a higher protein-to-fat ratio compared to other meats (OECD/FAO, 2025). Additionally, environmental considerations play a role in the shift toward poultry consumption, as red meat production is more resource-intensive and generates higher greenhouse gas emissions, making poultry a preferred choice for sustainability-conscious consumers (OECD/FAO, 2025).

At the level of poultry production, global meat output is projected to grow by 13 %, equivalent to 46 million tons carcass weight, reaching approximately 406 million tons carcass weight by 2034. Over half of this increase (55 %) is expected to occur in Asia, with poultry production rising by 15 million tons. China is projected to contribute nearly 10 % of the global growth in meat production, followed by India (8 %), the United States (8 %), and Vietnam (7 %) (OECD/FAO, 2025). Additionally, Latin America is anticipated to gradually expand its share of production, leveraging its competitive advantages in land availability, feed resources, and livestock genetics. Poultry is expected to maintain its dominant role in the meat sector, accounting for 62 % of the total increase in meat production over the coming decade (OECD/FAO, 2025). The fastest growth in poultry production is projected in upper-middle-income countries, driven by strong domestic demand. Poultry offers multiple advantages over other meat sources, including shorter production cycles, high feed conversion efficiency, lower overall production costs, and the ability to be raised near rapidly expanding urban markets (OECD/FAO, 2025).

Ultimately, poultry industry is a cornerstone of global food security, providing high-quality, affordable protein to millions of consumers and meeting both current and future nutritional needs amid population growth and socio-economic changes. Poultry accounts for approximately 40 % of total global meat production, with consumption projected to reach 145 million metric tons by 2030 and supply around 45 % of total meat-derived protein by 2034. The sector also supports environmental sustainability by reducing waste and greenhouse gas emissions, while its ease of preparation and quick cooking make it a healthy and convenient protein source. As a key driver of sustainable production, the poultry industry enhances food security, fosters economic and social development, and ensures a secure and sustainable food future.

### Technological advancements in the poultry industry enabling increased production efficiency and enhanced sustainability

Poultry industry has undergone profound technological advancements to meet the dual challenges of increasing global population and sustainability demands. Innovations in genetics, nutrition, housing, disease control, and digital management, including precision farming and artificial intelligence, have enhanced productivity, feed efficiency, and animal welfare. These developments enable the production of high-quality protein with reduced environmental impact, positioning poultry as a key sector in ensuring global food security and sustainable protein supply.

Over recent decades, the poultry industry has been profoundly transformed from traditional methods to integrated, technology-driven systems aimed at achieving comprehensive sustainability. Key advancements include enhanced genetic selection via genomic tools, adoption of digital technologies and artificial intelligence for precision farming and real-time monitoring of bird health and welfare, optimized feed formulation for cost-effective nutrition, and modern processing techniques improving meat quality while reducing manual labor (Castro et al., 2023). Together, these innovations underpin the current and future poultry sector, ensuring the production of safe, high-quality, and environmentally sustainable protein while maintaining optimal animal welfare and meeting global consumer demands (Castro et al., 2023). This is partly due to the fact that poultry consumption is not constrained by religious or cultural restrictions, unlike red meat. In addition, the prices of chicken meat and its products have remained relatively lower than those of beef, reflecting the growing poultry industry’s sustained ability to achieve high productivity and efficiency over the years (Tavárez and Solis de los Santos, 2016).

Globally, more than 66 billion broiler chickens are slaughtered annually. This enormous scale of poultry meat production makes welfare concerns widespread and likely to increase due to global population growth, rising meat demand, and the continued focus on production efficiency within the agricultural sector. Consequently, the commercial poultry industry represents one of the greatest challenges for animal welfare in modern agriculture (Hartcher and Lum, 2020). To meet this growing demand, the broiler industry aims to achieve high performance and efficiency in terms of body weight, feed conversion ratio, and meat yield, while maintaining optimal animal welfare (Honig et al., 2024). However, the expansion of the poultry sector is attributed to factors such as population growth, economic capacity, and urbanization trends. As a result, the poultry industry plays a central role in meeting global protein demand amid demographic shifts and changing lifestyles (Mottet and Tempio, 2017).

Since the 1950s, animal breeding has achieved significant advancements in improving livestock productivity. By the 1970s, the objectives of poultry breeding had broadened beyond a sole focus on production to include enhancing efficiency, reducing environmental impact, promoting animal health and welfare, improving food quality and safety, and conserving genetic diversity (Neeteson et al., 2023). These developments have been driven by intensive research and the adoption of modern selection techniques, which are expected to continue evolving in the future. With the ongoing global expansion of poultry meat production and consumption, the diversity of commercial crossbreeds is projected to grow further to meet varied market demands and consumer preferences. Coupled with improvements in animal welfare, environmental sustainability, and cost-efficiency, poultry remains well-positioned to supply healthy, lean animal protein to populations worldwide (Neeteson et al., 2023).

These developments are vividly reflected when comparing poultry meat production performance over the decades. For instance, in 1957, a 42-day-old broiler weighed approximately 586 grams with a feed conversion ratio (FCR) of 2.8 g feed/g body weight gain. Today, broilers of the same age weigh around 2,900 grams with an FCR below 1.70. This significant improvement is attributed to advances in production technologies that enhance performance, including improved genetics and breeding, better understanding of nutrition and feed management, and overall enhanced management practices. Genetic and breeding improvements account for about 80–90 % of the current production gains, emphasizing that optimal nutrition and environmental management are essential to fully realizing the bird’s genetic potential (Tavárez and de los Santos, 2016). Similarly, Zuidhof et al. (2014) demonstrated that broiler growth rates increased by more than 400 % between 1950 and 2005 when genetically representative birds from those years were raised under identical conditions (Tallentire et al., 2016). Additionally, broiler production has shown remarkable progress over recent decades. Between 1985 and 2010, the body weight and feed conversion ratio of broilers at 35 days improved from 1.4 to 2.4 kg and from 2.3 to 1.5, respectively (Siegel, 2014; Castro et al., 2023).

In recent years, broiler flocks have achieved an average live weight of 2.5 kg at 37.7 days of age, with FCR of 1.53. Intensive selection for improved FCR has produced highly feed-efficient birds, making poultry production more environmentally sustainable compared to many other meat sources. Since 1972, the amount of feed required per kilogram of body weight in broilers has decreased by 0.89 kg, representing a total reduction of 2.2 kg of feed for birds reaching 2.5 kg. These long-term gains correspond to an average of approximately 20 grams less feed per kilogram of live weight. Beyond the environmental advantages, enhanced FCR directly reduces the use of agricultural resources. For instance, an FCR improvement of −0.02 in a broiler system processing one million birds per week at a target weight of 2.5 kg leads to annual savings of approximately 2,600 tons of feed and 4,700 tons of water, while freeing up around 557 hectares of agricultural land, including areas used for maize, wheat, and soy production (Neeteson et al., 2023).

The notable improvements in broiler performance can be largely attributed to genetic advancements in these birds (Zuidhof et al., 2014), which also carry significant environmental implications. Feed provision represents one of the largest environmental impacts within the poultry industry (Prudêncio da Silva et al., 2014). Feed-efficient birds that require less feed to achieve the same slaughter weight directly reduce the industry’s environmental footprint through two main mechanisms: (1) lowering the environmental impacts associated with feed production, including greenhouse gas emissions from fossil fuel use in crop cultivation and land-use changes, and (2) reducing nutrient emissions from poultry manure, which are also lower in more feed-efficient birds. Therefore, studying the genetic traits influencing feed-use efficiency is essential for improving productivity while minimizing environmental impact (Tallentire et al., 2016). Additionally, the feed conversion ratio in broilers continues to improve by approximately two to three points per year (Avendaňo et al., 2017). By projection, a 2 kg broiler in 2025 will require about 300 grams less feed than it does today, a 10 % reduction, resulting in lower greenhouse gas emissions (Kleyn and Ciacciariello, 2021).

In this context, poultry industry has emerged as a cornerstone for global food security by providing a highly efficient, accessible, and sustainable source of high-quality animal protein. Through transformative advances in genetic selection, nutrition, housing, and precision management technologies, including artificial intelligence and real-time monitoring systems, the industry has dramatically enhanced production efficiency, growth rates, and feed conversion ratios while maintaining optimal animal welfare. These improvements have enabled the production of more protein with less feed, water, and land, thereby reducing the environmental footprint of poultry farming compared to alternative meat sources. With over 66 billion broilers produced annually, poultry delivers a reliable, affordable, and culturally unrestricted protein source, capable of meeting the nutritional needs of a rapidly growing global population. The cumulative effect of genetic, nutritional, and technological innovations ensures that poultry production not only supports increasing dietary demands but also strengthens sustainability by minimizing resource use, greenhouse gas emissions, and environmental impacts, positioning the sector as a strategic driver of both current and future food security worldwide.

The development of poultry vaccines has undergone a remarkable transformation over the past few decades, driven by advances in molecular biology and bioinformatics. These innovations have enabled the precise identification of antigenic targets and the design of multivalent vaccines that offer broader protection against emerging diseases while accelerating vaccine development (Abdelaziz et al., 2024). For instance, reverse vaccinology is now used to analyze the pathogen’s proteome and identify potential vaccine candidate proteins, whereas the integration of bioinformatics with immunogenetics has improved vaccine efficacy and stability compared to conventional approaches (Abdelaziz et al., 2024). Moreover, nanotechnology has shown great promise as a delivery system for mucosal vaccines in poultry, such as those targeting Infectious Bursal Disease Virus (IBDV) and Chicken Anemia Virus (CAV), despite challenges related to production costs. These advances address the ongoing challenges posed by the emergence and reemergence of infectious diseases, emphasizing the continued need to enhance vaccine efficacy, stability, and delivery methods to safeguard poultry health and productivity (Abdelaziz et al., 2024).

While genetic improvements in fast-growing poultry represent a qualitative leap in the poultry industry, contributing to rapid growth rates and enhanced feed conversion efficiency, broiler chickens can only fully express their genetic potential under optimal environmental conditions within the farm. Such conditions play a crucial role in maximizing productive performance, achieving consistent and optimal results, and ensuring healthy growth and welfare of fast-growing birds. Accordingly, just as a revolution has occurred in genetic advancements in broiler breeding, a parallel transformation has taken place in housing systems, shifting from traditional structures to technologically advanced, AI-integrated production environments.

The poultry industry has greatly benefited from progress in genetics, nutrition, housing systems, and management strategies. Modern high-yield poultry breeds tend to be healthier and more robust than those of three decades ago (Korver, 2023). The adoption of artificial intelligence in poultry production represents a pivotal advancement in the field, enhancing both production efficiency and animal welfare through intelligent systems for early disease detection and behavioral analysis. These systems enable precise environmental and nutritional management, contributing to more sustainable and higher-quality production models (Neethirajan, 2022).

In recent years, the poultry farming sector has witnessed a major shift toward large-scale, intensive, and smart production systems. The evolution of internet of things has facilitated the expansion of precision livestock farming (Zhao et al., 2025). Through interconnected electronic systems, it is now possible to centrally manage data from multiple farms, including environmental parameters, water and electricity consumption, data analytics, and production management, with high efficiency. These systems also help optimize environmental conditions within poultry houses by integrating acoustic analysis, environmental monitoring, and production data management with financial analytics tools. Collectively, these innovations provide comprehensive solutions that enhance the efficiency of poultry housing systems and support the integration of precision livestock farming models with advanced data analytics (Liu et al., 2024).

Ultimately, poultry industry has undergone a transformative evolution over the past decades, driven by synergistic advancements in genetics, nutrition, housing, management, and precision technologies. These developments have not only maximized production efficiency and growth performance but have also prioritized animal welfare, environmental sustainability, and food safety. The integration of artificial intelligence, real-time monitoring systems, and precision livestock farming has enabled producers to optimize resources, reduce environmental impacts, and respond proactively to emerging challenges, including disease outbreaks. As global demand for high-quality, affordable, and culturally unrestricted protein continues to rise, poultry production stands as a cornerstone of food security and a model of sustainable animal agriculture. Looking forward, the continued convergence of scientific innovation, technological integration, and strategic breeding programs will be essential to meet the nutritional needs of a growing population, strengthen the resilience of production systems, and ensure that poultry remains a reliable, sustainable, and ethically responsible source of animal protein worldwide.

### Consumer behaviors and preferences and their impact on poultry production

Currently, consumers have access to a wide and diverse range of products to meet their needs, marking the end of the era in which producers dominated the market and controlled consumer choices. In this new era, consumers hold the actual power, determining which products companies should produce (Toha and Supriyanto, 2023). Consumer behavior is defined as any activity or action influenced by multiple factors and associated with the efforts made to obtain products that satisfy their needs and desires. Understanding human behavior is highly complex, to the extent that fully expressing it in words is nearly impossible (Toha and Supriyanto, 2023). Consequently, analyzing consumer behavior forms the cornerstone of marketing management, as marketing strategies and plans must be built on a deep and precise understanding of the consumers who constitute a company’s target market (Toha and Supriyanto, 2023). Psychological factors that significantly influence consumer behavior include motivation, perception, attitudes, beliefs, and lifestyle. Understanding these elements contributes to a deeper comprehension of consumer behavior and helps identify the most effective ways to attract individuals toward making purchasing decisions (Wikansari et al., 2022).

In this context, poultry industry is directly shaped by consumer behaviors and preferences, which influence production patterns, product development, and marketing strategies. Growing awareness of health, ethics, and environmental sustainability has increased demand for high-quality, safe, and sustainable poultry products, driving technological innovation, improved nutrition, and enhanced animal welfare. Therefore, understanding consumer behavior serves as a strategic tool for producers and decision-makers to ensure demand fulfillment, optimize production efficiency, and balance profitability with sustainability.

Moreover, consumer preferences for poultry products are influenced by a combination of factors, such as price, the geographic origin of the product and feed, and rearing conditions, rather than relying on a single attribute. Distinct consumer segments emerge, differing in their sensitivity to economic and social dimensions. Accordingly, the successful introduction of new or alternative products into the market depends on focusing on the attributes that matter most to consumers, thereby enhancing acceptance and generating economic, social, and environmental benefits, such as supporting local production, promoting sustainability, and upholding ethical considerations in animal welfare (del Bosque et al., 2021). During economic crises, consumers may prefer cheaper raw products, whereas in periods of higher purchasing power, other criteria beyond price—such as environmental impact, animal welfare, and preference for local products become more significant, making local products more attractive. This trend may lead to increased poultry imports at the expense of domestic production (Jez et al., 2011).

Moreover, another increasingly strong consumer trend today is meat production with attention to animal welfare. The consumption of organic foods has become particularly popular among younger generations as part of a lifestyle focused on health and well-being (Statista, 2020). Poultry constitutes the vast majority of organic livestock in the European Union, numbering approximately 60 million birds (FIBL-IFOAM, 2022), with the highest organic chicken consumption recorded in 2018 in the United States (27.7 %) and the European Union (24.4 %) (Statista, 2019). Furthermore, consumers are increasingly focusing on the origin of meat and the methods used in rearing it according to animal welfare standards, prioritizing quality over quantity and shifting from fresh meat consumption to processed meat products (EC, 2018).

For example, in China, fresh food consumption channels are divided into traditional agricultural markets, supermarkets, e-commerce for fresh products, and other secondary channels. Traditional markets remain the primary channel for fresh food sales, accounting for 73 %, followed by supermarkets at 22 %, while e-commerce represents only about 3 % of the channels. Fresh food consumption increased notably from 312 million tons in 2014 to 332 million tons in 2018, with per capita consumption of fresh vegetables and fruits, meat and poultry, eggs, and dairy products rising continuously between 2013 and 2019. Consumption of fresh meat, including poultry, stands out as a major trend within this growth. Regarding e-commerce, more than 72 % of consumers purchase vegetables, 61.2 % buy fruits, while consumption of meat and poultry reaches 58.1 % (Sun, 2021). These data indicate that poultry constitutes a significant portion of fresh meat consumption among Chinese consumers, with a gradual increase in reliance on modern channels such as e-commerce.

In light of different age groups, white meats enjoy considerable popularity among young people as future consumers, who often avoid red meats for environmental, ethical, and animal welfare reasons (Viktória and Zoltán, 2023). Health awareness, price consciousness, and effort expectancy are also key factors shaping consumer attitudes toward locally produced chicken, particularly among young, educated, and health-conscious individuals who value both health benefits and affordability. Furthermore, product availability emerges as an important factor, highlighting the role of easy access to local chicken in consumer decision-making. Although environmental awareness does not directly impact attitudes or purchasing behavior, it reflects the complex nature of consumer choices. In contrast, effort expectancy directly influences purchasing behavior, emphasizing the importance of convenience and accessibility, especially when buying locally produced chicken (Abbasi et al., 2024).

Poultry industry is directly influenced by consumer preferences, with factors such as price, the geographic origin of the product and feed, rearing conditions, and the emphasis on organic products and animal welfare serving as key determinants of purchasing behavior, particularly among younger generations. This diversity gives rise to multiple consumer segments with varying sensitivities to economic and social dimensions, requiring producers to focus on the attributes most valued to ensure market acceptance, promote sustainability, support local production, and uphold ethical standards. Economic fluctuations also shape consumer priorities, as lower-priced products are favored during crises, while environmental criteria and local origin gain importance with increased purchasing power, compelling producers to adopt sustainable and transparent production practices, enhance rearing systems, and utilize smart technologies and advanced monitoring systems. Moreover, the growing demand for organic poultry and white meat highlights the need to innovate products that meet quality, health, and convenience expectations, while strengthening modern distribution channels such as e-commerce to ensure efficient product delivery. Simultaneously, evolving market trends, including demand for locally produced, fresh, and convenient products, have reshaped production and distribution strategies, creating both challenges and opportunities for the local and global poultry sectors. Therefore, understanding consumer behavior and preferences serves as a vital strategic tool for the poultry industry, enhancing producers’ capacity to meet future demand and achieve a sustainable balance between profitability, quality, and social responsibility.

Conversely, convenience, affordability, and perceived health benefits continue to be the primary factors driving poultry consumption. Consumers often favor poultry, particularly chicken breast, due to its ease of preparation and perceived nutritional advantages. In many cases, concerns about human health take precedence over ethical considerations related to animal welfare. Additionally, misconceptions stemming from distrust or unfamiliarity with regulations and labeling practices may limit acceptance of higher-priced products, while gaps in consumer knowledge can also influence purchasing decisions. Despite these challenges, the well-established reputation of poultry as a healthy, convenient, and affordable protein source remains the dominant factor underpinning its consumption. Understanding the attributes that consumers value, along with the motivations and barriers shaping their choices, is therefore essential for designing strategies that encourage the adoption of sustainable production practices. Enhancing consumer awareness of the sustainability of poultry production can further increase acceptance and willingness to bear the additional costs associated with sustainable practices (Dewi et al., 2024).

Additionally, the sustainability of poultry production depends on consumer demand and the perceptions that shape purchasing behavior. Estes et al. (2015) reported that consumers have expressed concerns about whether conventionally produced poultry contains unsafe levels of antibiotics or hormones, and organic poultry is often perceived as healthier than conventional poultry. Therefore, marketing and communication efforts should be designed to enhance consumer understanding of antibiotic use and the absence of hormones in poultry production, while also highlighting the health benefits of conventionally produced poultry. Moreover, marketing messages must accurately reflect actual conventional poultry production practices, and agricultural communication specialists should promote logical and critical thinking to counter campaigns that disseminate misleading information about farming (Estes et al., 2015).

Therefore, consumer awareness and preferences are pivotal in shaping the future of the poultry industry, with demand centered on convenience, affordability, and perceived health benefits, alongside growing attention to organic poultry and animal welfare. Knowledge gaps and misconceptions emerge as factors limiting product acceptance, necessitating that producers enhance transparency and adopt sustainable production practices. Moreover, increasing consumer understanding of production sustainability not only boosts product acceptance but also drives innovation and improvements in rearing and production methods, achieving an optimal balance between quality, profitability, and environmental and social sustainability.

Designer meats represent innovative poultry products developed to meet consumer demands for better taste, quality, and nutritional value while promoting health and ethical production practices. They are produced through genetic selection, advanced feeding strategies, and modern farming techniques, and are often enriched with omega-3 fatty acids, vitamins, antioxidants, and selenium using nutrient-rich ingredients such as flaxseed and fish oil. These products provide notable health benefits, including improved brain function and cardiovascular health, making them appealing to health-conscious consumers willing to pay premium prices for fortified foods that align with their dietary goals. However, their production is costly and complex, requiring precise management and consumer education about their advantages. Studies indicate that European consumers and younger, higher-income groups are more receptive to such functional foods, while older consumers primarily purchase them for their health benefits (Abbas et al., 2024).

With increasing education and health awareness, populations are increasingly favoring safe and healthy foods, and organic poultry occupies a prominent position in meeting this global demand, with its production expected to grow rapidly. Organic poultry meat is characterized by high market demand and premium prices due to specialized practices and the costs associated with organic production, while good production practices contribute to improved bird health and performance without compromising animal welfare (Yadav et al., 2022). Additionally, organically produced foods or those raised under natural agricultural systems hold strong market appeal for certain consumers due to health, environmental, animal welfare, and food sustainability considerations (Lay, Jr. et al., 2011). Organic poultry farming exemplifies environmentally sustainable production practices, relying on natural processes, minimizing industrial inputs, enhancing animal welfare, and maintaining ecosystem health (European Union, 1999; Bist et al., 2024).

Additional factors have also contributed to the economic growth of alternative agricultural products. For example, the increasing consumer interest in locally produced foods, particularly fresh products, has provided an extra incentive to seek out alternative agricultural goods and products from nearby farms (Feldmann and Hamm, 2015). Furthermore, the growing demand for convenient, ready-to-use products has led to the introduction of a wide range of fresh, cut-up poultry products and more extensively processed items, where shelf life plays a crucial role in consumer purchasing decisions, as products with longer shelf life are considered more convenient (Barbut and Leishman, 2022).

Poultry industry is dominated by major American and Brazilian companies that rely on global markets to secure supplies and focus on minimizing meat production costs. The high competitiveness of these global suppliers leaves limited room for maneuver for locally producing sectors. However, potential opportunities for local production exist, supported by the development of new relationships between producers and consumers, as well as changes in labeling and quality systems that ensure origin, freshness, production methods, and environmental impact of the products (Jez et al., 2011).

The poultry industry operates within an increasingly complex and interconnected global environment, where biological, economic, environmental, and social factors interact to create a wide range of risks and challenges that directly influence consumer behaviors and preferences. Effective management is required to ensure production continuity and sustainability, as well as to safeguard food security at both regional and global levels (Nassar and Abbas, 2025b). Consumer confidence, product quality and safety, product diversity, and the emergence and re-emergence of diseases are key factors affecting purchasing decisions and consumer trends, which in turn shape poultry production patterns and marketing strategies within the sector (Hafez et al., 2020).

The COVID-19 pandemic exemplifies how sudden global events can significantly impact consumer behavior, leading to notable changes in poultry demand and supply chains, despite the fact that chickens are not susceptible to SARS-CoV-2 infection (Hafez et al., 2020). During the pandemic, widespread rumors linking the consumption of chicken and eggs to COVID-19 transmission in humans caused severe disruptions in consumer behavior. For instance, the poultry industry in India experienced a sharp decline in consumption, demand, and prices, necessitating rapid intervention by health authorities to mitigate misinformation and ensure continued consumer purchase of chicken (Kolluri et al., 2021; Kathiravan, 2024).

The pandemic also affected consumer behavior regarding pricing and preference for local products. Egg prices rose significantly during lockdowns as consumers adjusted their purchasing patterns, which in turn had a substantial impact on international poultry trade during this period (Hafez et al., 2021). Notably, these behavioral shifts were more pronounced in developing countries, where reliance on imported essential inputs such as feed, vaccines, medicines, and equipment exacerbated economic pressures and challenged the ability to meet local consumer demand (Attia et al., 2022).

This experience demonstrates that consumer behaviors and preferences, whether driven by health concerns, misinformation, or economic considerations, directly and decisively affect poultry production and market strategies. It underscores the critical importance for producers to understand and analyze consumer behavior to ensure sustainable production and respond effectively to changing demand in a complex global environment.

In light of recent developments, future trends in the poultry industry point toward a focus on healthy and functional products, such as organic poultry meat with enhanced nutritional value and health benefits to meet the growing demand from health-conscious consumers. Environmental sustainability and animal welfare are expected to become core priorities, with production practices that minimize industrial inputs, rely on natural processes, and maintain bird and ecosystem health. Poultry science programs will continue to drive innovation through specialized breeding, advanced feeding strategies, and intelligent production systems, addressing evolving market preferences for fresh, cut-up, and ready-to-cook products while ensuring freshness and shelf life. Despite the dominance of major global companies, opportunities for local production exist through strengthening producer-consumer relationships and improving labeling and quality systems to guarantee origin, production methods, and environmental impact. Additionally, digital technologies and smart monitoring systems will play an increasingly important role in optimizing productivity, tracking bird health, and analyzing demand, contributing to cost reduction and efficiency. Altogether, these developments are set to make the poultry sector more innovative, responsive to consumer trends, environmentally sustainable, and economically efficient.

### Strategic role of poultry science programs in shaping the future of global food security and achieving sustainability

Food security and sustainability are among the most pressing challenges facing the world in the twenty-first century, driven by rapid population growth, ongoing urbanization, climate change, and increasing pressures on natural resources. In this context, the poultry industry occupies a strategic position as a highly efficient source of animal protein, capable of providing effective and timely solutions to meet societal nutritional needs. However, ensuring that poultry production systems contribute sustainably and effectively requires more than technological innovation; it necessitates a comprehensive understanding of consumer behavior and the integration of education and capacity-building programs. The interaction between poultry production sciences and consumer preferences represents a pivotal nexus for shaping flexible and sustainable food systems, as advances in breeding, nutrition, management, and biotechnology must align with evolving market demands, ethical standards, and environmental sustainability principles to ensure the production of food products that are both healthy and socially and environmentally responsible.

On the other hand, poultry science programs extend beyond traditional academic instruction, serving as a strategic cornerstone for a sustainable food future by integrating scientific research, practical training, and consumer insights. By equipping professionals with the necessary skills, knowledge, and innovative mindset, these programs enable the implementation of effective and sustainable production strategies. Furthermore, they contribute to fostering innovation, enhancing the resilience of food systems, and supporting evidence-based policymaking, thereby establishing themselves as a critical component in building an integrated and sustainable global food system.

The current study proposes a framework structured around six interrelated pillars that form the foundation for the strategic role of poultry production sciences in shaping the future of global food security and achieving sustainability. These pillars are as follows:

#### Engine for innovation and sustainable production

Poultry science programs represent a fundamental pillar within the agricultural innovation ecosystem, serving as living laboratories for generating scientific and applied solutions that contribute to the development of advanced production strategies. These strategies include improving genetic lines to enhance productivity and quality, increasing feed conversion efficiency through smart feeding systems based on precise and bio-informed nutritional analysis, and integrating rearing techniques that prioritize animal welfare while reducing stress and disease incidence. Such innovations enable the production of high-quality poultry meat using fewer resources and lower carbon emissions, thereby enhancing sector competitiveness and supporting global trends toward environmentally balanced and sustainable livestock production.

To effectively fulfill this role, poultry science programs must adopt an integrated approach that combines education, research, and field application. This involves updating curricula to incorporate concepts of sustainability and digital innovation, developing research laboratories equipped with artificial intelligence and Internet of Things technologies for real-time monitoring of biological performance, and establishing strategic partnerships with production facilities and agricultural companies to implement research outcomes in practice. Additionally, fostering a culture of agricultural entrepreneurship among students and encouraging innovative projects in sustainable poultry production is essential. Programs should also develop environmental assessment frameworks to monitor resource efficiency and reduce carbon footprints, enabling them to lead the transition toward sustainable production aligned with global Sustainable Development Goals.

#### Bridging research and consumer behavior

Poultry science programs play a strategic role in creating a robust bridge between scientific research and consumer behavior by conducting in-depth analyses of market data and understanding evolving consumer preferences. Their scope extends beyond technical aspects of production to include studies on purchasing motivations, perceptions of product quality and safety, and the factors influencing dietary decisions. Through these analyses, the programs can anticipate future market needs and design production and marketing strategies that align closely with consumer demands at both local and global levels. This insight also guides the industry toward developing innovative poultry products, such as organic meat, antibiotic-free options, or nutritionally enhanced products, thereby increasing consumer trust and driving greater adoption of sustainable and healthy food choices.

To effectively fulfill this role, poultry science programs must adopt a multidisciplinary approach that integrates animal production science, food marketing, data analytics, and consumer psychology. This requires establishing specialized research units focused on market and consumer behavior, and training students and researchers in statistical analysis and artificial intelligence tools to identify behavioral patterns and predict demand trends. Collaboration with food companies and marketers is also essential to translate research outcomes into new products that meet public health requirements and global quality standards. In doing so, poultry science programs become an effective link between research laboratories and consumers’ tables, supporting industry sustainability and reinforcing the concept of knowledge- and demand-driven production.

#### Developing specialized human capital

Poultry science programs serve as a cornerstone in building the human capital necessary to lead the future of modern poultry production. These programs go beyond preparing graduates with specialized scientific knowledge; they aim to cultivate professionals who combine technical competence, analytical skills, and applied innovation. By integrating theoretical learning with hands-on training in real production environments, students acquire advanced skills in managing modern farms, operating smart rearing systems, and analyzing production data to develop strategies for enhancing performance and product quality. Additionally, students gain opportunities to design innovative poultry products that meet evolving market demands while applying principles of circular economy and environmental sustainability in resource management. Moreover, it should be emphasized that poultry science programs are designed not only to meet the workforce needs of the industry but also to cultivate graduates who are socially responsible and aware. By combining practical training with professional values, analytical skills, and applied innovation, these programs ensure that students acquire the competencies required in the poultry sector while fostering awareness of societal responsibilities and sustainable practices.

To effectively achieve this role, poultry science programs must adopt an integrated educational and training approach that bridges academia and industry. This includes updating curricula to incorporate future-oriented skills such as digital transformation, big data analytics, and agricultural entrepreneurship. Strengthening partnerships with production facilities and food companies to provide practical field training is essential for preparing students to face real-world industry challenges. Furthermore, investing in faculty members with practical and industrial experience, alongside establishing innovation incubators and consultancy centers that link research with market needs, ensures the development of a generation of specialized leaders capable of connecting industrial production with scientific innovation and delivering sustainable, high-quality products that satisfy consumer expectations.

#### Community education and awareness

Poultry science programs play a pivotal role in raising public awareness about the importance of transitioning toward healthy and sustainable dietary practices by leveraging academic knowledge to serve society. These programs promote a culture of nutritional awareness through interactive workshops, educational campaigns, and applied research projects that link responsible production with informed consumption. The initiatives aim to highlight the nutritional value of poultry products, emphasize the benefits of organic and antibiotic-free poultry, and demonstrate the role of sustainable production practices in protecting the environment and reducing carbon emissions. Additionally, these programs help communities better understand the concept of food security and the importance of supporting local products that adhere to sustainability and quality standards.

To effectively fulfill this role, poultry science programs should adopt a comprehensive community engagement strategy that builds strong bridges between universities and local communities. This includes developing digital interactive platforms to disseminate knowledge on food safety and sustainability, as well as organizing field initiatives targeting schools, small farms, and production entities to promote best practices in poultry rearing and consumption. Integrating students into community awareness activities as part of graduation requirements further enhances their sense of social responsibility and provides practical experience in disseminating scientific knowledge. Through this approach, poultry science programs become an influential community force, fostering more conscious and sustainable consumption behaviors while contributing to both national and global food security.

#### Policy support and decision-making

Poultry science programs serve as a pivotal component in supporting decision-making and shaping policies related to sustainable food production. They function as scientific advisory centers, providing evidence-based insights and accurate data to policymakers and regulatory authorities. Through applied research and field studies, these programs offer reliable information on best practices for production management, efficiency enhancement, and ensuring the quality and safety of poultry products. They also contribute to the development of regulatory frameworks that promote the prudent use of resources, minimize waste and emissions, and enhance the sector’s competitiveness at both national and international levels. Moreover, the outputs of these programs help establish comprehensive policies for consumer protection, enforcement of health and safety standards, and balancing economic growth with environmental sustainability.

To effectively fulfill this strategic role, poultry science programs should strengthen partnerships with government agencies and the private sector to exchange knowledge and provide scientific consultations based on the latest research. This includes establishing specialized research units to analyze agricultural and food policies and developing national databases documenting performance, production, and consumption indicators. Programs can also organize conferences and workshops that bring together researchers and policymakers to share expertise and discuss future sectoral challenges. Through this integration of scientific research and decision-making, poultry science programs become an intellectual driving force that guides national policies toward achieving sustainable food security and enhancing the country’s position in the global agricultural economy.

Moreover, universities should engage responsibly in policy development while maintaining scientific integrity and academic objectivity. They can provide evidence-based recommendations, support the development of regulatory frameworks, and guide decisions related to sustainable food production and food security, all while preserving the independence of scientific research. By balancing policy advocacy with academic neutrality, universities can raise public awareness, train students in critical and leadership thinking, and prepare professionals capable of effectively implementing sustainable policies. In this way, poultry science programs become strategic platforms that integrate scientific research, practical education, and community engagement to support policymaking and advance sustainable development and food security at both local and global levels.

#### Accelerating social and economic transformation

Poultry science programs play a pivotal role in accelerating social and economic transformation by linking production systems with markets and consumer behavior, thereby contributing to rural development and promoting local production. Their impact extends beyond the development of advanced production methods, serving as intellectual and research platforms that help reshape traditional food systems into more integrated and sustainable models. By fostering a deep understanding of the relationship between production and consumption, these programs contribute to building flexible food supply chains capable of adapting to social and economic changes while simultaneously meeting the growing demand for healthy, environmentally friendly, and locally sourced products. Furthermore, they support agricultural entrepreneurship and social innovation by encouraging small- and medium-sized enterprises in sustainable poultry production and marketing, positively influencing rural development and job creation.

To effectively fulfill this transformative role, poultry science programs must adopt a holistic approach that integrates scientific research, practical education, and community engagement. This includes designing curricula that incorporate agricultural economics, food sociology, and digital marketing within animal production sciences. Strengthening partnerships with local development institutions and international organizations is also essential for implementing transformative projects that combine economic sustainability with social equity. Through this integrated approach, poultry science programs become a driving force for change, contributing to the development of modern food systems that reflect future social transformations and support a knowledge-based, sustainable, and innovative national economy.

Additionally, implementation of the proposed framework for poultry science programs is essential to maximize their strategic impact on global food security and sustainability. This framework organizes efforts across six interrelated pillars, innovation and sustainable production, bridging research and consumer behavior, developing specialized human capital, community education and awareness, policy support, and accelerating social and economic transformation, providing a comprehensive vision of the role of poultry science programs within the global food system. Its distinctiveness lies in the integration of scientific research, practical training, and understanding of market and consumer needs, alongside fostering partnerships with both government and private sectors, enabling the translation of knowledge into effective and sustainable practical applications. The framework also ensures the development of specialized human competencies, the innovation of environmentally friendly production methods and products, and the support of evidence-based decision-making. Moreover, implementing this framework opens new horizons for future research and studies by identifying unexplored scientific and technological areas, stimulating the development of innovative solutions aligned with evolving global challenges, thereby reinforcing the role of poultry science programs as a driving force for academic, technological, and societal advancement.

## Conclusion

Poultry industry plays a strategic role in achieving global food security, particularly in light of the current and projected rapid population growth. The poultry meat sector has undergone profound transformations, shifting from raising dual-purpose birds for both meat and egg production to developing specialized meat breeds that grow faster and exhibit higher productivity. At the market level, the focus has moved from selling whole birds to marketing cut-up and further processed products, aligning with the needs of modern consumers. Concurrently, consumer behavior has evolved significantly, with a preference for pre-cut and ready-to-cook products due to convenience and preparation speed, while also emphasizing nutritional quality and freshness. New preferences have emerged for organically or naturally produced products, alongside increasing demand for packaged, ready-to-cook items, with critical considerations such as shelf life and product purity, making consumers a key driving force shaping production and marketing trends in the sector. Technological advancements have also enhanced production inputs, including housing, feeding tools, and water supply, alongside improvements in genetic efficiency, feed conversion ratios, and vaccination programs to prevent diseases, thereby maintaining the sector’s productivity.

In this context, poultry science programs play a strategic and pivotal role in enhancing global food security and promoting sustainability by integrating scientific research, practical training, and an understanding of consumer behaviors and preferences. These programs contribute to the development of innovative and sustainable production through improved genetic lines, smart feeding strategies, and modern rearing systems that prioritize animal welfare and reduce carbon emissions. They also cultivate specialized human capital capable of innovation, managing smart farms, supporting evidence-based policymaking, and raising community awareness of sustainable practices. Overall, these programs enable the sector to translate knowledge into innovative and sustainable production practices that enhance competitiveness, meet future demand, and position poultry science as a transformative driver for a sustainable and innovative poultry industry.

## Funding

This work was supported by the Deanship of Scientific Research, Vice Presidency for Graduate Studies and Scientific Research, King Faisal University, Saudi Arabia (Grant No. KFU253737).

## CRediT authorship contribution statement

**Farid S. Nassar:** Writing – review & editing, Writing – original draft, Visualization, Validation, Supervision, Software, Resources, Project administration, Methodology, Investigation, Funding acquisition, Formal analysis, Data curation, Conceptualization.

## Disclosures

I hereby formally declare, as the sole author, that there is no conflict of interest.

## References

[bib0001] Abbas G., Arshad M., Imran M. (2024). Consumer preferences and market trends: customizing poultry products for customer-based poultry markets. Pak. J. Sci..

[bib0002] Abbasi I.A., Shamim A., Ashari H. (2024). Factors influencing consumers’ purchase behavior toward indigenous chicken: insights from a cognitive affect behavior model. Br. Food J..

[bib0003] Abdelaziz K., Helmy Y.A., Yitbarek A. (2024). Advances in poultry vaccines: leveraging biotechnology for improving vaccine development, stability, and delivery. Vaccines.

[bib0004] Ashrafudoulla M., Na K.W., Byun K.-H. (2021). Isolation and characterization of Salmonella spp. From food and food contact surfaces in a chicken processing factory. Poult. Sci..

[bib0005] Attia Y.A., Rahman M.T., Hossain M.J. (2022). Poultry production and sustainability in developing countries under the COVID-19 crisis: lessons learned. Animals.

[bib0006] Avendaňo S., Neeteson A., Fancher B. (2017). Proceedings of the Poultry Beyond 2023 Conference, New Zealand.

[bib0007] Barbut S., Leishman E.M. (2022). Quality and process ability of modern poultry meat. Animals.

[bib0008] Bist R.B., Bist K., Poudel S. (2024). Sustainable poultry farming practices: a critical review of current strategies and future prospects. Poult. Sci..

[bib0009] Bowen G.A. (2009). Document analysis as a qualitative research method. Qual. Res. J..

[bib0010] Castro F.L.S., Chai L., Arango J. (2023). Symposium^:^ poultry industry paradigms: connecting the dots. J. Appl. Poult. Res..

[bib0011] Chowdhury M.A.H., Ashrafudoulla M.d., Mevo S.I.U. (2023). Current and future interventions for improving poultry health and poultry food safety and security: A comprehensive review. Compr. Rev. Food Sci. Food Saf..

[bib0012] Conrad Z., Niles M.T., Neher D.A. (2018). Relationship between food waste, diet quality, and environmental sustainability. PLoS ONE.

[bib0014] del Bosque C.I.E., Spiller A., Risius A. (2021). Who wants chicken? Uncovering consumer preferences for produce of alternative chicken product methods. Sustainability.

[bib0015] Dewi G., Smith C., Martin W. (2024). Focus groups exploring American consumer perspectives on contemporary poultry production reveal critical insights to educate sustainable practices for producers. Front. Sustain. Food Syst..

[bib0016] EC (2018). https://ec.europa.eu/info/food-farming-flsheries/farming/facts-and-flgures/markets/outlook/medium-term_hu.

[bib0017] EC (2021).

[bib0018] EC (2022).

[bib0019] Estes S., Edgar L.D., Johnson D.M. (2015). Consumer perceptions of poultry production: a focus on Arkansas. J. Appl. Commun..

[bib0020] European Union (1999). COUNCIL REGULATION (EC) No 1804/1999. https://www.legislation.gov.uk/eur/1999/1804/pdfs/eur_19991804_1999-08-24_en.pdf.

[bib0021] FAO, 2022. Food Outlook - Biannual Report on Global Food Markets. Rome, Italy. doi:10.4060/cb9427en.

[bib0022] FAO. 2021. World Food and Agriculture - Statistical Yearbook 2021. Rome. Italy, pp, 1–368. doi:10.4060/cb4477en.

[bib0023] FAO (2023). Production | gateway to poultry production and products | food and agriculture organization of the United Nations. https://www.fao.org/poultry-productionproducts/production/en/.

[bib0024] Feldmann C., Hamm U. (2015). Consumers’ perceptions and preferences for local food: a review. Food Qual. Pref..

[bib0025] FlBL-lFOAM (2022). The world of organic agriculture statistics and emerging trends 2022. https://www.fibl.org/.fileadmin/documents/shop/1344-organic-world-2022_lr.pdf.

[bib0026] Foley J., Ramankutty N., Brauman K. (2011). Solutions for a cultivated planet. Nature.

[bib0027] Hafez H.H., Attia Y.A., Bovera F. (2021). Influence of COVID-19 on the poultry production and environment. Environ. Sci. Pollut. Res..

[bib0028] Hafez H.M., Attia Y.A. (2020). Challenges to the poultry industry: current perspectives and strategic future after the COVID-19 outbreak. Front. Vet. Sci..

[bib0029] Hartcher K.M., Lum H.K. (2020). Genetic selection of broilers and welfare consequences: a review. World’s Poult. Sci. J..

[bib0031] Honig H., Haron A., Plitman L. (2024). Comparative analysis of broiler Housing systems: implications for production and wellbeing. Animals.

[bib0032] Jez C., Beaumont C., Magdelaine P. (2011). Poultry production in 2025: learning from future scenarios. World’s Poult. Sci. J..

[bib0033] Kathiravan G. (2024). Analysing determinants of household broiler chicken meat purchases amidst Social-Media misinformation: a Tobit study. Agro Productividad.

[bib0034] Kleyn F.J., Ciacciariello M. (2021). Future demands of the poultry industry: will we meet our commitments sustainably in developed and developing economies?. World's Poult. Sci. J..

[bib0035] Kolluri G., Tyagi J.S., Sasidhar P.V.K. (2021). Research note: Indian poultry industry vis- `a-vis coronavirus disease 2019: a situation analysis report. Poult. Sci..

[bib0013] Korver D.R., R D. (2023). Review: current challenges in poultry nutrition, health, and welfare. Animal.

[bib0036] Lambert V.A., Lambert C.E. (2012). Qualitative descriptive research: an acceptable design. Pac. Rim Int. J. Nurs. Res..

[bib0037] Lay D.C., Fulton R.M., Hester P.Y. (2011). Hen welfare in different housing systems. Poult. Sci..

[bib0038] Liu M., Chen H., Zhou Z. (2024). Development of an intelligent service platform for a poultry house facility environment based on the internet of things. Agriculture.

[bib0039] Mottet A., Tempio G. (2017). Global poultry production: current state and future outlook and challenges. World’s Poult. Sci. J..

[bib0040] Nassar F.S., Abbas A.O. (2025). Digital twin in poultry production: A framework for enhancing workforce to job market readiness. Poult. Sci..

[bib0041] Nassar F.S., Abbas A.O. (2025). Crisis and risk management in poultry production: preparing future leaders for sustainability and food security. Poult. Sci..

[bib0044] Neeteson A.-M., Avendaño S., Koerhuis A. (2023). Evolutions in commercial meat poultry breeding. Animals.

[bib0045] Neethirajan S. (2022). Automated tracking systems for the assessment of farmed poultry. Animals.

[bib0046] Neuman W.L. (2014).

[bib0047] OECD/FAO (2021).

[bib0048] OECD/FAO, 2023. OECD-FAO Agricultural Outlook 2023-2032, Paris, France, pp, 1–359. doi:10.1787/08801ab7-en.

[bib0049] OECD/FAO, 2025, OECD-FAO Agricultural Outlook 2025-2034, Paris and Rome, pp, 1–166. doi:10.1787/601276cd-en.

[bib0050] OECD/FAO (2022).

[bib0051] Patton M.Q. (2002).

[bib0052] Prudêncio da Silva V., van der Werf H.M.G., Soares S.R. (2014). Environmental impacts of French and Brazilian broiler chicken production scenarios: an LCA approach. J. Environ. Manag..

[bib0053] Siegel P.B. (2014). Evolution of the modern broiler and feed efficiency. Annu. Rev. Anim. Biosci..

[bib0054] Statista (2019). Global organic chicken consumption. https://www.statista.com/statistics/975681/organic-chicken-consumption-by-region-worldwide/.

[bib0055] Statista (2020). Meat trends in Europe. A statista dossier plus on meat industry trends and the future of meat in Europe. https://www.statista.com/study/70192/meat-trends-in-europe.

[bib0056] Sun X. (2021). International Conference on Tourism, Economy and Environmental Sustainability (TEES 2021), Fujian, China.

[bib0057] Tallentire C.W., Leinonen I., Kyriazakis I. (2016). Breeding for efficiency in the broiler chicken: a review. Agron. Sustain. Dev..

[bib0058] Tavárez M.A., Solis de los Santos F. (2016). Impact of genetics and breeding on broiler production performance: a look into the past, present, and future of the industry. Anim. Front..

[bib0059] Toha M., Supriyanto (2023). Factors influencing the consumer research process: market target, purchasing behavior, and market demand (literature review of consumer behavior). Post Mod. Econ. J..

[bib0061] United Nations, 2022. World Population Prospects 2022: Summary of Results. New York, USA.

[bib0060] United Nation (2021). Population, food security, nutrition and sustainable development. United Nations - department of economic and social affairs. https://www.un.org/development/desa/dpad/wp-content/uploads/sites/45/publication/PB_102.pdf.

[bib0062] United Nations (2019). 9.7 billion on Earth by 2050, but growth rate slowing, says new UN population report | UN News. https://news.un.org/en/story/2019/06/1040621.

[bib0063] USDA (2025). Livestock and poultry: World Markets and Trade. https://apps.fas.usda.gov/psdonline/circulars/livestock_poultry.pdf.

[bib0064] Viktória V., Zoltán S. (2023). Analysis of consumer behaviour in the European poultry meat market. Appl. Stud. Agribus. Commer..

[bib0065] Weber R.P. (1990).

[bib0066] Wikansari R., Ausat R.A., Al Hidayat R. (2022). Proceedings of the International Conference on Economic, Management, Business and Accounting, ICEMBA 2022, 17 December 2022, Tanjungpinang, Riau Islands, Indonesia.

[bib0067] Yadav S.P.S., Ghimire N.P., Yadav B. (2022). Key requirements, status, possibilities, consumer perceptions, and barriers of organic poultry farming: A review. Fund. Appl. Agric..

[bib0068] Zhao H., Lilin Y., Zhichao H. (2025). Error analysis strategy for long-term correlated network systems: generalized nonlinear stochastic processes and dual-layer filtering architecture. IEEE Internet of Things J.

[bib0069] Zuidhof M.J., Schneider B.L., Carney V.L. (2014). Growth, efficiency, and yield of commercial broilers from 1957, 1978, and 2005. Poult. Sci..

